# Supplementary material for: Individual response to antidepressants for depression in adults-a meta-analysis and simulation study
Source: PLoS One. 2020 Aug 27;15(8):e0237950. doi: 10.1371/journal.pone.0237950 (PMC7451660; doi:10.1371/journal.pone.0237950)
Supplement: S2 Checklist — (PDF) [file pone.0237950.s002.pdf]

PRISMA 2009 Flow Diagram

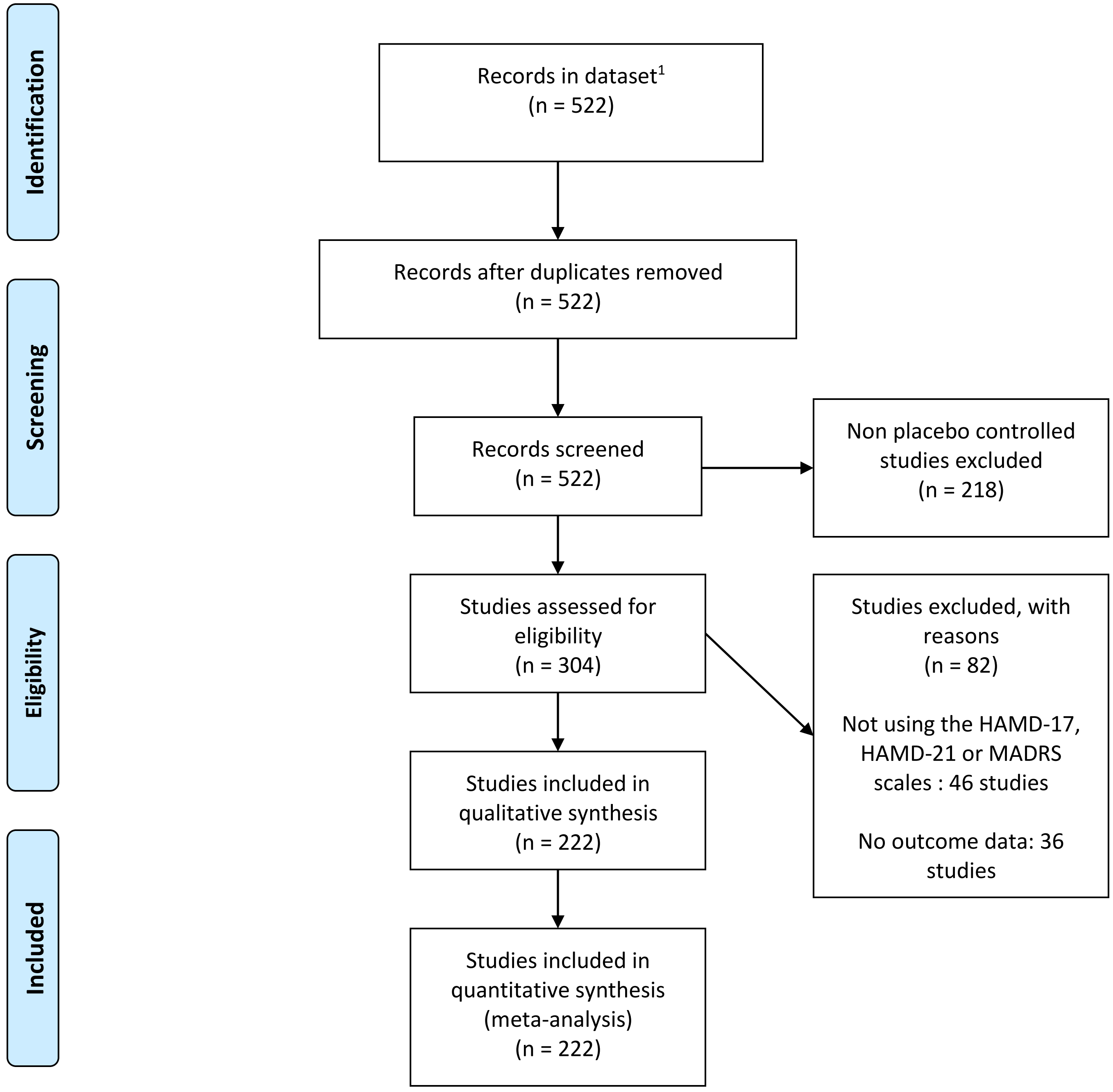

References

1. Cipriani A, Furukawa TA, Salanti G, et al. Comparative efficacy and acceptability of 21 antidepressant drugs for the acute treatment of adults with major depressive disorder: a systematic review and network meta-analysis. *Lancet.* 2018;391(10128):1357-1366.

From: Moher D, Liberati A, Tetzlaff J, Altman DG, The PRISMA Group (2009). *P*referred *R*eporting *I*tems for *S*ystematic Reviews and *M*eta-*A*nalyses: The PRISMA Statement. PLoS Med 6(7): e1000097. doi:10.1371/journal.pmed1000097

For more information, visit www.prisma-statement.org.
