# Supplementary material for: Individual response to antidepressants for depression in adults-a meta-analysis and simulation study
Source: PLoS One. 2020 Aug 27;15(8):e0237950. doi: 10.1371/journal.pone.0237950 (PMC7451660; doi:10.1371/journal.pone.0237950)
Supplement: S1 Protocol — (PDF) [file pone.0237950.s003.pdf]

ADMINISTRATIVE INFORMATION

TITLE
Individual response to antidepressants for depression in adults – a simulation study and meta-analysis.

Identification
Protocol.

REGISTRATION
The finalized protocol will be registered on OSF/PsyArXiv.

AUTHORS
Klaus Munkholm, M.D., DMSc.[1,*]
Stephanie Winkelbeiner, Ph.D.[2]
Philipp Homan, M.D., Ph.D.[2]

Contact information
[1] Nordic Cochrane Centre, Rigshospitalet, Copenhagen, Denmark.
[2] Psychiatric University Hospital Zurich, University of Zurich, Zurich, Switzerland

* Corresponding author:
Klaus Munkholm, M.D., DMSc.
Nordic Cochrane Centre
Rigshospitalet, Dept. 7811
Blegdamsvej 9
DK-2100 Copenhagen
Denmark
E-mail: km@cochrane.dk
Telephone: +4535457112

Contributions
KM, SW and PH conceived the study. KM wrote the first draft of the protocol. All authors critically revised the protocol for important intellectual content.

SUPPORT

Sources
The authors report no funding for the study.

INTRODUCTION

RATIONALE

Personalized medicine concerns the matching of the individual to a given therapy that best matches them and the condition in question. This implies that a certain treatment will be right for one individual while another treatment will be right for another individual, both suffering from the same condition. The underlying assumption is that the response to treatment differs between individuals.

The efficacy of a drug is usually tested in randomized controlled trials, which derive an average effect of the drug across the participants in the trial. A common interpretation of such trials is that those responding to the treatment, often labeled “responders” differ from those not responding, similarly labeled “non-responders”, both then assumed to be permanent features of the individual. An alternative interpretation is that individual response to treatment cannot be predicted in that the participants are indistinguishable from each other with regard to response to treatment, the differences in response reflecting random variation (Senn, 2004). Randomized controlled trials can, however, not distinguish between these scenarios – that of individual response treatment or random variability or any scenario in between (Senn, 2004).

In depression research, efforts motivated by the prospects of personalized antidepressant treatment, have been ongoing for many years (Holsboer, 2008), investigating the potential for pharmacogenetic markers (Boland, Duffy, & Myer, 2018), other biomarkers (Cattaneo et al., 2016; Jha & Trivedi, 2018) and clinical characteristics (Green et al., 2017) to inform treatment. The expectation is that it will be possible to identify biological markers to identify patients that will “respond” to antidepressants. Thus, although there is uncertainty regarding antidepressants’ potential beneficial effects and their harms due to limitations in their evidence base (Munkholm, Paludan-Muller, & Boesen, 2019), the small apparent effect found in meta-analyses (Cipriani, Furukawa, et al., 2018) is believed to be the result of some patients experiencing substantial benefit, while others may have no benefit of the treatment (Carter et al., 2012; Cipriani, Salanti, et al., 2018). There is, however, little reason to assume that a drug that appears to be marginally effective in a larger population would perform otherwise in a subpopulation (Senn, 2018) or even in certain individuals. Although evidence suggests few differences between antidepressants’ apparent effects (Cipriani, Furukawa, et al., 2018), the consequence for the individual patient of the assumption of individual differences in treatment response may be a trial-and-error process, that involves treatment being given with different antidepressants in succession if no improvement is observed, to identify a treatment that is potentially effective for that particular patient (Leuchter, Cook, Hunter, & Korb, 2009; Schuyler, 2009).

Estimating individual response to treatment, what is referred to as the treatment-by-patient interaction, is complex and requires non-RCT study designs such as repeated crossover trials (Senn, 2016). Although RCTs are thus not capable of distinguishing individual response to treatment from other components of variation, they may, however, provide some information regarding the potential presence of individual treatment response through observing the difference in variation in the response to the treatment between the intervention group and the control group (Senn, 2016). An increase in the variance in the treatment group compared with the control group could indicate the presence of variation in response to treatment and a treatment-by-patient interaction (Cortes et al., 2018; Senn, 2016). This method involves computing the ratio between

the variance in the treatment group and the control group – the variability ratio. A ratio greater than 1, indicating higher variance in the treatment group than the control group, could indicate the presence of a differing individual response to the treatment (Nakagawa et al., 2015). Recently, this method was used to investigate whether evidence from randomized controlled trials of antipsychotics for schizophrenia indicated a treatment-by-patient interaction (Winkelbeiner, Leucht, Kane, & Homan, 2019). That study found a slightly lower variance in the treatment group compared with the control group, suggesting no personal element of response to treatment (Winkelbeiner et al., 2019). Here, wish to extend that work to investigate whether the variances in the treatment response are indicative of an individual response to treatment with antidepressants for depression in adults, while also providing an illustration of the different components of variation in RCTs.

OBJECTIVES

We wish to a) illustrate the different components of variation in randomized controlled trials (RCTs), highlighting the treatment-by-patient interaction as the component of interest, using simulated data and b) investigate the empirical evidence of a treatment-by-patient interaction by conducting a meta-analysis of the variability ratio in RCTs of antidepressants for depression in adults.

METHODS

TRIAL SIMULATION

To illustrate the different components of variation in RCTs we will simulate data from adult patients with depression who were randomized to either treatment with antidepressants or placebo and assessed the severity of depressive symptoms using the 17-item Hamilton Depression Rating Scale (HAMD-17) and found a positive effect of antidepressants. We will use an RCT of sertraline versus placebo for adult depression (Schneider et al., 2003) to inform the simulation parameters and will use a sample size of 15 participants in each group.

We will first simulate the data to illustrate the distribution of effects among all participants and how the dichotomization of the treatment group into categories of “responders” and “non-responders” based on arbitrary thresholds can generate a perception of individual differences in response to treatment.

Second, we will simulate the addition of a single crossover condition with a) a constant treatment effect across individuals and b) a treatment effect varying across individuals to illustrate that ranking participants according their observed outcome on a continuous scale is a flawed approach to quantifying symptom improvement.

Third, we will simulate the repeated measurement over time in four patients to illustrate random within-patient variation.

Fourth, we will simulate, adding a double crossover condition, to illustrate how only such a design can separate the random within-participant variation from the treatment-by-patient interaction, which is the component reflecting the individual differences in treatment response.

META-ANALYSIS

For the meta-analysis of the variability ratio, we will use the data included in a recent meta-analysis of antidepressants for depression in adults, comprising 522 studies and 116.477 participants (Cipriani, Furukawa, et al., 2018). The authors of the meta-analysis searched the Cochrane Central Register of Controlled Trials, CINAHL, Embase, LILACS database, MEDLINE, MEDLINE In-Process, PsycINFO, AMED, the UK National Research Register, and PSYNDEX from the date of their inception to Jan 8, 2016, with no language restrictions, supplemented with manual searches for published, unpublished, and ongoing RCTs in international trial registers, websites of drug approval agencies, and key scientific journals in the field (Cipriani, Furukawa, et al., 2018). They included double-blind RCTs comparing antidepressants (provided dosing was within the therapeutic range) with placebo or another antidepressant as oral monotherapy for the acute treatment of adults (≥18 years of age, both sexes) with a primary diagnosis of major depressive disorder (Cipriani, Furukawa, et al., 2018). They included all second-generation antidepressants approved by the regulatory agencies in the USA, Europe, or Japan and, additionally, included the two tricyclics amitriptyline and clomipramine included in the WHO Model List of Essential Medicines and lastly also trazodone and nefazodone, because of their “distinct effect and tolerability profiles” (Cipriani, Furukawa, et al., 2018).

The data is available online, accompanying the published article (Cipriani, Furukawa, et al., 2018) (https://data.mendeley.com/datasets/83rthbp8ys/2).

ELIGIBILITY CRITERIA

For our study, we will apply the following eligibility criteria:

We will include placebo-controlled studies only.

Studies that reported change or endpoint scores on the 17-item Hamilton Depression Rating Scale (HAMD) (Hamilton, 1960), the 21-item HAMD or the Montgomery Åsberg Depression Rating Scale (MADRS) (Montgomery & Asberg, 1979), which were the most used scales among the studies (Munkholm et al., 2019), will be included, provided they reported the necessary information (mean, standard deviation and sample size).

STUDY RECORDS

Data management

We will download the full online dataset (https://data.mendeley.com/datasets/83rthbp8ys/2) and import it into the statistical software R.

Selection process

Eligible studies will be selected based on the information provided in the online dataset, using R code.

Data collection process

We will not collect additional data beyond that available in the online dataset.

DATA ITEMS

We will use the following information from the studies: study identification (e.g. first author, trial registration number), study year, mean baseline to endpoint change score or endpoint score (and standard deviation) on the 17-item HAMD, 21-item HAMD and the MADRS scales, respectively, and sample size.

DATA SYNTHESIS

We will describe the study sample by summary statistics of the number of included studies, number of included participants, the various antidepressants investigated in the included studies and the depression symptom severity scales used.

Planned quantitative synthesis

The SDs of the baseline to endpoint change-scores include the same variance components in the treatment group and control group, respectively. The treatment group may, additionally, contain a potential treatment-by-patient interaction, which could indicate individual differences in the treatment response. A higher variance in the treatment group compared with the control group would thus indicate a potential variable treatment effect.

To assess this variance, we will calculate for each study the relative variability of the antidepressant treatment group and the placebo control group as the log variability ratio (log VR) (Hedges & Nowell, 1995) using the formula

$$logVR = log\left(\frac{SD_{tx}}{SD_{ct}}\right) + \frac{1}{2(N_{tx}-1)} - \frac{1}{2(N_{ct}-1)}$$

where SD_{tx} is the reported sample SD for the treatment group, sd_{ct} the reported sample SD for the control group, N_{tx} the treatment sample size and N_{ct} the control sample size (Nakagawa et al., 2015). We will further calculate the corresponding sampling variance, SD^2_{logVR}, for each comparison between antidepressant drugs and placebo using the formula

$$SD^2_{logVR} = \frac{1}{2(N_{tx}-1)} - \frac{1}{2(N_{ct}-1)}$$

The log VR may be limited in its applicability in situations where there is dependence between the mean and the variance. We will therefore check for such a mean-variance relationship by fitting a linear model with the SDs for the baseline to endpoint change scores as the dependent variable and the mean of the baseline to endpoint changes scores as the predictor with the sample size as weight, for the treatment group and control group, respectively. In case of an association between the mean and the variance, we will calculate the log coefficient of variation ratio (log CVR) as an additional effect size statistic for investigating variability differences between the two treatment groups (Nakagawa et al., 2015). Log CVR can be expressed as

$$logCVR = log\left(\frac{CV_{tx}}{CV_{ct}}\right) + \frac{1}{2(Ntx-1)} - \frac{1}{2(Nct-1)}$$

where CV_{tx} and CV_{ct} are $SD_{tx}/\bar{x}_{tx}$ and $SD_{ct}/\bar{x}_{ct}$, respectively.

We will weigh log VR or log CVR values with the inverse of its corresponding sampling variance (Viechtbauer, 2010) and enter it into a random-effects model, which allows for the quantification of the true individual response, after adjusting for within-patient variability and regression to the mean (Cortes et al., 2018; Hecksteden et al., 2015). We will back-transform the results, which produces a variability ratio, which, for values greater than 1, will indicate greater variability under treatment with antidepressants compared with placebo, and for values below 1 will indicate less variability under treatment compared with the control condition.

Where multiple treatment arms were investigated in the same study, we will not combine the treatment arms but divide the sample size of the placebo group by the number of treatment arms while retaining the mean and SD, creating multiple pair-wise comparisons for each study (Higgins, Green, & Cochrane Collaboration., 2008). Our primary outcome will be the overall summary estimate for the variability ratio across all included comparisons. We will conduct subgroup analyses according to the specific antidepressant drugs, the depression symptom severity scale used in the study and whether the outcome was baseline to endpoint change scores or endpoint scores.

The analyses will be performed in R.

REFERENCES

Boland, J. R., Duffy, B., & Myer, N. M. (2018). Clinical utility of pharmacogenetics-guided treatment of depression and anxiety. *Personalized Medicine in Psychiatry, 7-8*, 7-13. doi:10.1016/j.pmip.2017.11.001

Carter, G. C., Cantrell, R. A., Victoria, Z., Haynes, V. S., Phillips, G., Alatorre, C. I., . . . Marangell, L. B. (2012). Comprehensive review of factors implicated in the heterogeneity of response in depression. *Depress Anxiety, 29*(4), 340-354. doi:10.1002/da.21918

Cattaneo, A., Ferrari, C., Uher, R., Bocchio-Chiavetto, L., Riva, M. A., Consortium, M. R. C. I., & Pariante, C. M. (2016). Absolute Measurements of Macrophage Migration Inhibitory Factor and Interleukin-1-beta mRNA Levels Accurately Predict Treatment Response in Depressed Patients. *Int J Neuropsychopharmacol, 19*(10). doi:10.1093/ijnp/pyw045

Cipriani, A., Furukawa, T. A., Salanti, G., Chaimani, A., Atkinson, L. Z., Ogawa, Y., . . . Geddes, J. R. (2018). Comparative efficacy and acceptability of 21 antidepressant drugs for the acute treatment of adults with major depressive disorder: a systematic review and network meta-analysis. *Lancet*. doi:10.1016/S0140-6736(17)32802-7

Cipriani, A., Salanti, G., Furukawa, T. A., Egger, M., Leucht, S., Ruhe, H. G., . . . Geddes, J. R. (2018). Antidepressants might work for people with major depression: where do we go from here? *The Lancet Psychiatry, 5*(6), 461-463. doi:10.1016/s2215-0366(18)30133-0

Cortes, J., Gonzalez, J. A., Medina, M. N., Vogler, M., Vilaro, M., Elmore, M., . . . Cobo, E. (2018). Does evidence support the high expectations placed in precision medicine? A bibliographic review. *F1000Res, 7*, 30. doi:10.12688/f1000research.13490.5

Green, E., Goldstein-Piekarski, A. N., Schatzberg, A. F., Rush, A. J., Ma, J., & Williams, L. (2017). Personalizing antidepressant choice by sex, body mass index, and symptom profile: An iSPOT-D report. *Personalized Medicine in Psychiatry, 1-2*, 65-73. doi:https://doi.org/10.1016/j.pmip.2016.12.001

Hamilton, M. (1960). A rating scale for depression. *J Neurol Neurosurg Psychiatry, 23*, 56-62.

Hecksteden, A., Kraushaar, J., Scharhag-Rosenberger, F., Theisen, D., Senn, S., & Meyer, T. (2015). Individual response to exercise training - a statistical perspective. *J Appl Physiol (1985), 118*(12), 1450-1459. doi:10.1152/japplphysiol.00714.2014

Hedges, L. V., & Nowell, A. (1995). Sex differences in mental test scores, variability, and numbers of high-scoring individuals. *Science, 269*(5220), 41-45. doi:10.1126/science.7604277

Higgins, J. P. T., Green, S., & Cochrane Collaboration. (2008). *Cochrane handbook for systematic reviews of interventions. Version 5.1.0 [updated March 2011].* Available from www.cochrane-handbook.org.

Holsboer, F. (2008). How can we realize the promise of personalized antidepressant medicines? *Nature Reviews Neuroscience, 9*, 638. doi:10.1038/nrn2453

Jha, M. K., & Trivedi, M. H. (2018). Personalized Antidepressant Selection and Pathway to Novel Treatments: Clinical Utility of Targeting Inflammation. *Int J Mol Sci, 19*(1). doi:10.3390/ijms19010233

Leuchter, A. F., Cook, I. A., Hunter, A. M., & Korb, A. S. (2009). A new paradigm for the prediction of antidepressant treatment response. *Dialogues Clin Neurosci, 11*(4), 435-446.

Montgomery, S. A., & Asberg, M. (1979). A new depression scale designed to be sensitive to change. *British Journal of Psychiatry, 134*(4), 382-389.

Munkholm, K., Paludan-Muller, A. S., & Boesen, K. (2019). Considering the methodological limitations in the evidence base of antidepressants for depression: a reanalysis of a network meta-analysis. *BMJ Open, 9*(6), e024886. doi:10.1136/bmjopen-2018-024886

Nakagawa, S., Poulin, R., Mengersen, K., Reinhold, K., Engqvist, L., Lagisz, M., . . . O'Hara, R. B. (2015). Meta-analysis of variation: ecological and evolutionary applications and beyond. *Methods in Ecology and Evolution, 6*(2), 143-152. doi:10.1111/2041-210x.12309

Schneider, L. S., Nelson, J. C., Clary, C. M., Newhouse, P., Krishnan, K. R., Shiovitz, T., . . . Sertraline Elderly Depression Study, G. (2003). An 8-week multicenter, parallel-group, double-blind, placebo-controlled study of sertraline in elderly outpatients with major depression. *Am J Psychiatry, 160*(7), 1277-1285. doi:10.1176/appi.ajp.160.7.1277

Schuyler, D. (2009). Trial and error. Retrieved from https://www.latimes.com/archives/la-xpm-2009-aug-03-he-depression-drug-choice3-story.html

Senn, S. (2004). Individual response to treatment: is it a valid assumption? *BMJ, 329*(7472), 966-968. doi:10.1136/bmj.329.7472.966

Senn, S. (2016). Mastering variation: variance components and personalised medicine. *Stat Med, 35*(7), 966-977. doi:10.1002/sim.6739

Senn, S. (2018). Statistical pitfalls of personalized medicine. *Nature, 563*(7733), 619-621. doi:10.1038/d41586-018-07535-2

Viechtbauer, W. (2010). Conducting Meta-Analyses inRwith themetaforPackage. *Journal of statistical software, 36*(3). doi:10.18637/jss.v036.i03

Winkelbeiner, S., Leucht, S., Kane, J. M., & Homan, P. (2019). Evaluation of Differences in Individual Treatment Response in Schizophrenia Spectrum Disorders: A Meta-analysis. *JAMA Psychiatry*. doi:10.1001/jamapsychiatry.2019.1530
