# Supplementary material for: Individual response to antidepressants for depression in adults-a meta-analysis and simulation study
Source: PLoS One. 2020 Aug 27;15(8):e0237950. doi: 10.1371/journal.pone.0237950 (PMC7451660; doi:10.1371/journal.pone.0237950)
Supplement: S1 File — (PDF) [file pone.0237950.s004.pdf]

Individual response to antidepressants for depression in adults – a meta-analysis and simulation study

Klaus Munkholm, M.D., DMSc.[1], Stephanie Winkelbeiner, Ph.D.[2], & Philipp Homan, M.D., Ph.D.[2]

[1] Nordic Cochrane Centre, Rigshospitalet, Copenhagen, Denmark

[2] University Hospital of Psychiatry Zurich, University of Zurich, Zurich, Switzerland

Author Note

The article, data and code are available online (https://osf.io/5gpe4/).

Correspondence concerning this article should be addressed to Klaus Munkholm, M.D., DMSc., Nordic Cochrane Centre, Rigshospitalet, Dept. 7811, Blegdamsvej 9, DK-2100 Copenhagen, Denmark. E-mail: km@cochrane.dk

Abstract

This Supplement accompanies the article - Individual response to antidepressants for depression in adults – a meta-analysis and simulation study.

Individual response to antidepressants for depression in adults – a meta-analysis and simulation study

Methods

Deviations from the protocol

We planned to investigate, as a secondary outcome measure, the coefficient of variation ratio (CVR), based on an assessment of the relationship between the means and standard deviations in the dataset. However, during the process of conducting the study, others raised attention to the fact that the correlation between the mean and SD from individual trials is not necessarily indicative of the CVR or whether the CVR differs between arms of the trial [1]. Where a relationship between the mean and the SD is observed, it may primarily be a reflection of a skewed distribution of the data [2]. Additionally, after the planning of the study we became aware that the depression severity scales used in the dataset did not have the characteristics of a ratio scale (see discussion in the main paper). As CVR analyses requires the underlying data to be measured on a ratio scale [1], we omitted CVR analyses.

Results

Simulation study

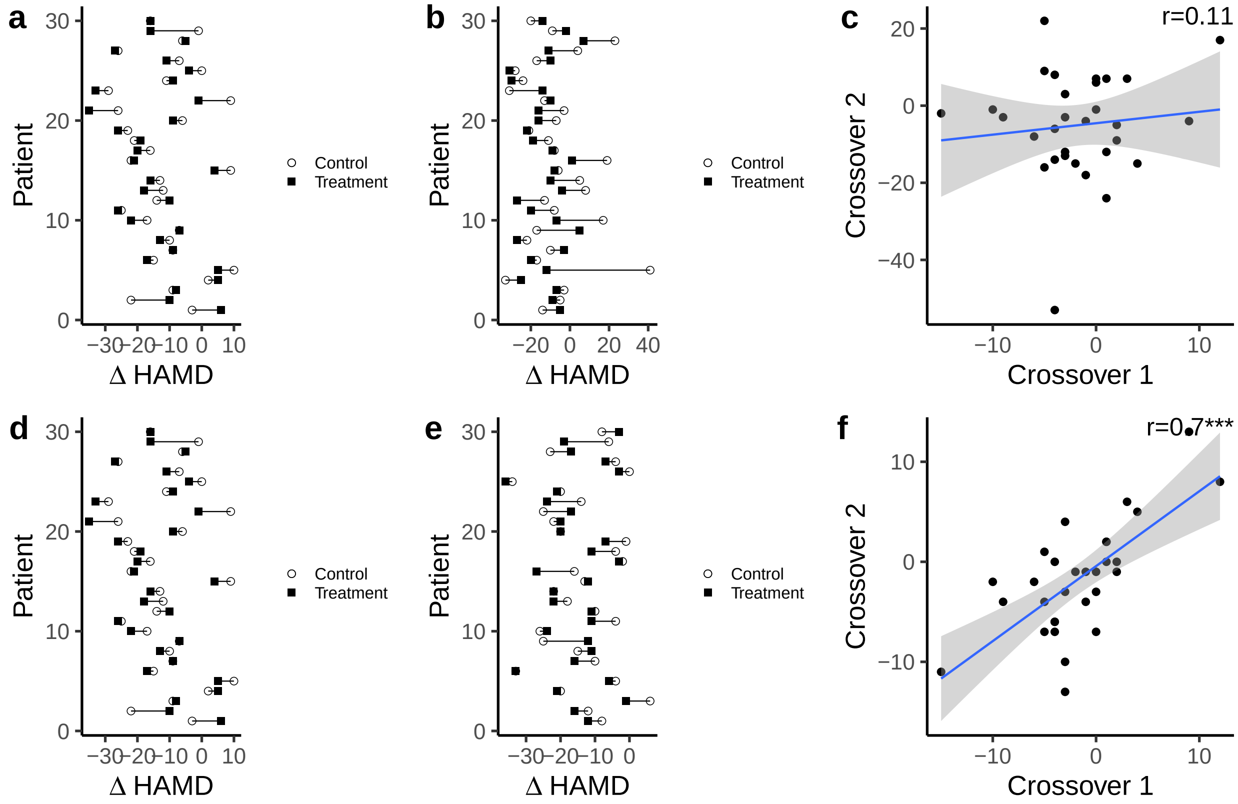

Figure 1. Repeated crossover trials are necessary to identify an individual treatment response that is a permanent characteristic of the individual. When adding a second crossover trial it is possible, that the effect observed in the initial cross-over trial (a), is not replicated in the subsequent crossover trial (b), indicating that the variation in effect is not moderated by the individual participant (c). Only if the effect observed in one crossover trial (d) is mirrored in a second crossover trial (e, f), can a treatment-by-patient interaction be separated from random within-patient variation and the presence of permanent individual differences in the response to treatment be determined.

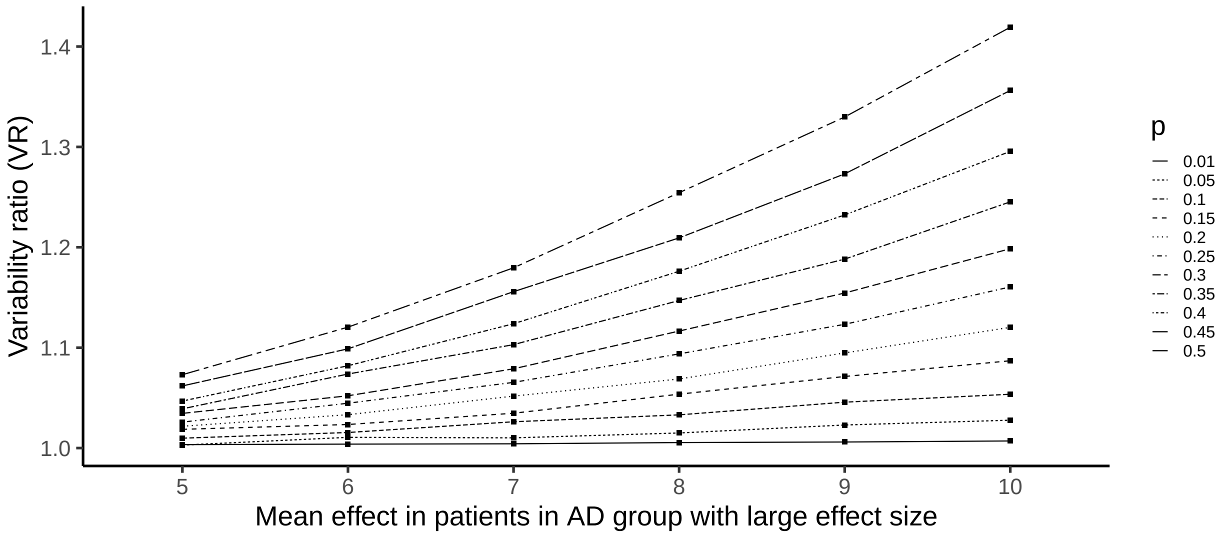

Figure 2. Simulation of 1000 trials of antidepressants versus placebo with 300 patients in each group, with an average effect of 2 (6) points on the HAMD-17. We simulated multiple scenarios with increasing effects of antidepressants in increasing proportions of the patients randomized to antidepressants. We assumed a normal distribution for the effects, but the true distribution, given individual responses, could have other shapes.
p: proportion of patients in the antidepressant group with a larger effect of antidepressants than the average 2 points on the HAMD-17; AD: antidepressant.

Meta-analysis of empirical data

Table 1
Mean and SD of outcomes.

Outcome type	Variable	HAMD17	HAMD21	MADRS
Change score	Outcome control group	-8.76 (1.92)	-8.65 (2.29)	-12.88 (1.46)
Change score	Outcome treatment group	-10.9 (2.1)	-11.17 (2.26)	-15.7 (1.82)
Change score	SD control group	7.54 (0.89)	8.32 (1.24)	10.05 (1.24)
Change score	SD treatment group	7.63 (1.11)	8.42 (1.27)	10.05 (1.18)
Endpoint score	Outcome control group	14.44 (3.46)	17.39 (3.33)	19.41 (2.82)
Endpoint score	Outcome treatment group	12.51 (2.2)	13.83 (2.62)	16.33 (2.71)
Endpoint score	SD control group	7.96 (1.47)	8.72 (1.44)	9.86 (0.87)
Endpoint score	SD treatment group	7.76 (1.29)	8.43 (1.11)	10.75 (1.39)

HAMD: Hamilton Depression Rating Scale; MADRS: Montgomery-Åsberg Depression Rating Scale; SD: standard deviation.

Table 2

Random effects meta-analysis of depression symptom severity outcomes.

Scale	Mean difference	95% CI
HAMD17	-1.9	-2.1; -1.7
HAMD21	-2.6	-3; -2.2
MADRS	-3.0	-3.6; -2.4

HAMD: Hamilton Depression Rating Scale; MADRS: Montgomery-Åsberg Depression Rating Scale; SD: standard deviation; CI: confidence interval.

Table 3

Indication of skew by group.

Group	Skewness	N
Antidepressant	No skew	11
Antidepressant	Skew	4
Antidepressant	Suggestive skew	89
Placebo	No skew	32
Placebo	Skew	1
Placebo	Suggestive skew	42

Indication of skew in studies with endpoint scores. Skewness was estimated by calculating the ratio of the mean divided by the standard deviation; A ratio less than 2 suggests skew, a ratio less than 1 is strong evidence of a skewed distribution. N: number of arms.

Table 4

VR subgroup analysis by depression symptom severity scale for endpoint scores.

Scale	Estimate	95% CI	I^2	Comparisons	N
HAMD17	0.98	0.95; 1.01	0.0	53	11120
HAMD21	0.96	0.92; 1	0.0	45	4724
MADRS	1.02	0.95; 1.11	1.5	6	1426
Total	0.98	0.96; 1	0.0	104	17270

HAMD: Hamilton Depression Rating Scale: MADRS, Montgomery-Åsberg Depression Rating Scale; SD: standard deviation; CI: confidence interval; Estimate: variability ratio.

Table 5

VR subgroup analysis by by depression symptom severity scale for change scores.

Scale	Estimate	95% CI	I^2	Comparisons	N
HAMD17	1.00	0.98; 1.02	0.00	113	21279
HAMD21	1.01	0.98; 1.04	13.77	85	12316
MADRS	1.00	0.97; 1.03	0.00	43	10279
Total	1.00	0.99; 1.02	0.00	241	43874

HAMD: Hamilton Depression Rating Scale; MADRS: Montgomery-Åsberg Depression Rating Scale; SD: standard deviation; CI: confidence interval; Estimate: variability ratio.

References

1. Mills HL, Higgins JPT, Morris RW, Kessler D, Heron J, Wiles N, et al. Detecting heterogeneity of intervention effects using analysis and meta-analysis of differences in variance between arms of a trial. 2020;

2. Bland JM, Altman DG. Transforming data. BMJ [Internet]. 1996;312(7033):770. Available from: https://www.ncbi.nlm.nih.gov/pubmed/8605469

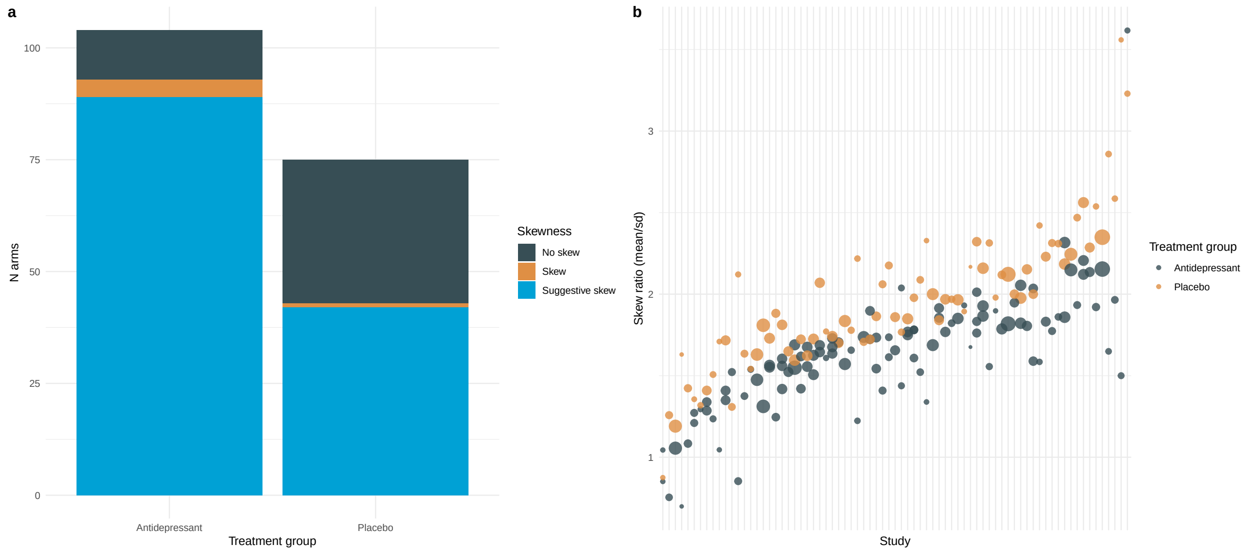

Figure 3. Potential indication of skew assessed from the means and standard deviations of endpoint outcome scores. Skewness was estimated by calculating the ratio of the mean divided by the standard deviation; A ratio less than 2 suggests skew, a ratio less than 1 is strong evidence of a skewed distribution. a) Indicators of skew in study arms. b) Ratio of mean/sd in the included studies. N: number of arms.

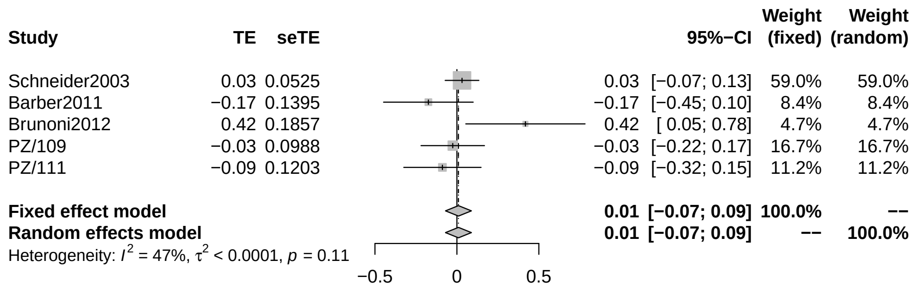

Figure 4. Forest plot of the log VR for sertraline versus placebo for endpoint scores.
In the subgroup analysis by antidepressant, heterogeneity was moderate for sertraline ($I^2 = 46.9\%$). We subsequently conducted a meta-analysis for sertraline alone. In this analysis the study Brunoni 2012 had a moderate log VR of 0.42. After excluding that study, the effect of larger heterogeneity in the sertraline group vanished ($VR = 0.99$, 95% CI: 0.91; 1.07, $I^2 = 0\%$).
I^2: inconsistency (%); CI: confidence interval, TE: log VR, seTE: standard error of the log VR.

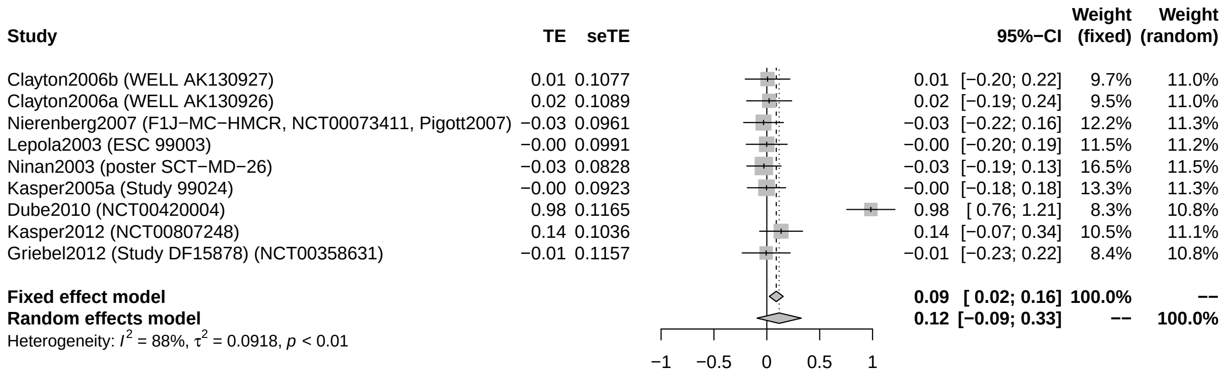

Figure 5. Forest plot of the log VR for escitalopram versus placebo for change scores.
In the subgroup analysis by antidepressant, heterogeneity was moderate for sertraline (I^2 = 87.9%). We conducted a meta-analysis for escitalopram alone. In this analysis the study Dube 2010 had a very large log VR of 0.98. After excluding that study, the effect of larger heterogeneity in the escitalopram group dissapeared (VR = 1.01, 95% CI: 0.94; 1.08, I^2 = 0%).
I^2: inconsistency (%); CI: confidence interval, TE: log VR, seTE: standard error of the log VR.
