# Supplementary material for: Individual response to antidepressants for depression in adults-a meta-analysis and simulation study
Source: PLoS One. 2020 Aug 27;15(8):e0237950. doi: 10.1371/journal.pone.0237950 (PMC7451660; doi:10.1371/journal.pone.0237950)
Supplement: S2 File — (PDF) [file pone.0237950.s005.pdf]

Individual response to antidepressants for depression in adults – a meta-analysis and simulation study. List of included studies

Klaus Munkholm, M.D., DMSc.[1], Stephanie Winkelbeiner, Ph.D.[2], & Philipp Homan, M.D., Ph.D.[2]

[1] Nordic Cochrane Centre, Rigshospitalet, Copenhagen, Denmark

[2] University Hospital of Psychiatry Zurich, University of Zurich, Zurich, Switzerland

Author Note

The article, data and code are available online (https://osf.io/5gpe4/).

Correspondence concerning this article should be addressed to Klaus Munkholm, M.D., DMSc., Nordic Cochrane Centre, Rigshospitalet, Dept. 7811, Blegdamsvej 9, DK-2100 Copenhagen, Denmark. E-mail: km@cochrane.dk

Abstract

This Supplement accompanies the article - Individual response to antidepressants for depression in adults – a meta-analysis and simulation study.

Individual response to antidepressants for depression in adults – a meta-analysis and simulation study. List of included studies

See appendix in Cipriani et al.[1] for complete reference citations.

1. Cipriani A, Furukawa TA, Salanti G, et al. Comparative efficacy and acceptability of 21 antidepressant drugs for the acute treatment of adults with major depressive disorder: A systematic review and network meta-analysis. *Lancet.* 2018;391(10128):1357-1366. doi:10.1016/S0140-6736(17)32802-7

ID
CL3-20098-022
CL3-20098-023
Kennedy2006 (CL3-20098-043)
Olie2007 (CL3-20098-042)
Stahl2010 (CAGO178A2302)
Zajecka2010 (CAGO178A2301) (NCT00411099)
CL3-20098-024
CL3-20098-026
CL3-20098-070
CAGO178A2303 (NCT00463242)
Loo2002 (CL2-014)
Kennedy2014 (EudraCT 2009-011238-84, CL3-20098-069)
Heun2013 (lSRCTl\l57507360)
Georgotas1982
Shipley1981
Roffman1982
Claghorn1983
Hormazabal1985
Rickels1985
Amsterdam1986
Paykel1988
Carman1991
Katz1993a
Katz1993b
Wilcox1994

(continued)

ID
Mynors-Wallis1995
Feighner1984
Lineberry1990 (WELL 84A)
Reimherr1998 (WELL203, FDA203)
Settle1999 (WELL212)
Coleman2001 (AK1A4007)
Tomarken2004
Clayton2006b (WELL AK130927)
Clayton2006a (WELL AK130926)
Hewett2009 (WXL101497)
Hewett2010a (AK130940) (NCT00093288)
Hewett2010b (AK130939)
Wellbutrin 06
GSK14
845
WELL 029
Wellbutrin 25
Study 205 (FDA) (WELL 205)
WELL AK1A4006
AK1102365
Koshino2013 (NCT01138007)
Study 89306 (FDA)
Montgomery1992 (Study 89303 FDA)
Feighner1999 (Study 91206 FDA)

(continued)

ID
Stahl2000
Liebowitz2008 (Study 332, NCT00277823)
Boyer2008 (Study 333, NCT00300378)(EUCTR2005-005463-28, 3151A1-333-EU)
Lieberman2008a (Study 309)(EUCTR2004-000562-13, 3151A1-309-EU, NCT00090649)
Lieberman2008b (Study 317)
Tourian2009 (NCT00384033)
Dunlop2011 (NCT00824291)
Clayton2013 (NCT01121484)
Iwata2013 (NCT00798707)
Liebowitz2013 (NCT00863798)
Clayton2015
DeMartinis2007 (Study 306, NCT00072774)
Goldstein2002 (HMAQ - Study Group A)
Study F1J-MC-HMAQ - Study Group B (starting page in the pdf document: 147)
Goldstein2004a (HMAT - Study Group A, ID#4091)
Goldstein2004b (HMAT - Study Group B, ID#4091)
Detke2002a (HMBH - Study Group A)
Detke2002b (HMBH - Study Group B)
Detke2004 (HMAY Study Group A)
Raskin2007 (HMBV) (NCT00062673)
Nierenberg2007 (F1J-MC-HMCR, NCT00073411, Pigott2007)
Perahia2006 (HMAY - Study Group B)
Higuchi2009
Cutler2009

(continued)

ID
NCT01145755
Lepola2003 (ESC 99003)
Ninan2003 (poster SCT-MD-26)
Wade2002 (ESC Study 99001 - FDA)
Kasper2005a (Study 99024)
Hirayasu2011a
Hirayasu2011b
Dube2010 (NCT00420004)
Kasper2012 (NCT00807248)
Griebel2012 (Study DF15878) (NCT00358631)
Mischoulon2014 (NCT00101452)
Study 19 (FDA) (Fabre1985)
Study 25 (FDA) (Rickels1986)
Study 62a (FDA) - MILD depression (Dunlop1990)
Study 62b (FDA) - MODERATE depression
Stark1985 (Study 27 - FDA)
Feighner1989a
Heiligenstein1994
Lam1995
Tollefson1995
McGrath2000
LopezRodriguez2004
NCT01808612
Fava2005

(continued)

ID
Bjerkenstedt2005
Moreno2005
Norton1984
Claghorn1996
Fabre1996
Montgomery2013 (F02695LP202, EudraCT2006-002404-34)
Gommoll2014 (LVM-MD-02, NCT00969150)
Asnis2013 (LVM-MD-01, NCT00969709)
Sambunaris2014 (LVM-MD-03, NCT01034462)
Bakish2014 (LVM-MD-10, NCT01377194)
MIR 003-003 (FDA)
MIR 003-020 (FDA)
MIR 003-021 (FDA)
Vartiainen1994 (MIR 84023 FDA)
MIR 84062 (FDA)
Smith1990 (MIR 003-024 FDA)
Bremner1995(MIR 003-022 - FDA)
Claghorn1995 (MIR 003-002 (FDA)
Halikas1995 (MIR 003-023 - FDA)
Kinoshita2009
003-048
003-008 (FDA)
003-042
D'Amico1990 (FDA 030-A2-0007)

(continued)

ID
Feighner1998
Fontaine1994 (FDA 03A0A-003)
Mendels1995 (FDA 03A0A-004B)
Rickels1994 (FDA CN104-005)
Rickels1995 (FDA CN104-006-1)
Cohn1996 (FDA CN104-006-2)
CN104-002 (FDA)
03A0A-004A (FDA)
PAR 01 001 (GSK & FDA)
Dunbar1993a (Claghorn1992, Rickels1989, Rickels1992, PAR 02-001 - FDA)
Dunbar1993b (Claghorn1992, PAR 02-002 - FDA)
Dunbar1993c (Smith1992, PAR 02-003 - FDA)
Feighner1993a (Feighner 1989c PAR 03 001 - FDA)
Feighner1993b (Cohn1990 Cohn1992 PAR 03 002 - FDA)
Feighner1993c (PAR 03 003 - FDA)
Feighner1993d (Shrivastava1992 PAR 03 004 - FDA)
Feighner1993e (Peselow1989 PAR 03 005 - FDA)
Feighner1993f (Fabre1992 PAR 03 006 - FDA)
29060 07 001
Dunbar1993d (Kiev1992, PAR 02-004 - FDA)
Miller1989 (MDUK/29060/III/82/006 (PAR-274) PAR UK 06 - FDA)
MY-1045/BRL-029060/1 (PAR 128)
MY-1043/BRL-029060/115
Dunner1992 (PAR 29060.09)

(continued)

ID
Edwards1989 (MD/PAR/009 PAR-276)
Golden2002a (29060/448)
Golden2002b (29060/449)
Rapaport2003 (PAR487)
Fava1998
Kramer1998
Jefferson2000 (29060/785)
Trivedi2004 (29060/810)
NKD20006 (NCT00048204)
Rapaport2009 (BRL-29060/874) (NCT00067444)
Higuchi2011 (PCR112810, NCT00866294)
Learned2012b Study2 (NCT00420641)
Keller2006c (Study062) (NCT00048607)
Griebel2012b (Study DF15879) (NCT00361491)
Versiani2000 (Study 091)
Study 032a (CTN032-FCE20124)
Study 015
Bosc1997a (Study 014 - Andreoli2002)
Study 049
Study 045
Clayton2003 (Study 050)
M/2020/0046 (Study 046)
M/2020/0047 (Study 047)
Studie009 (CTN009-FCE20124)

(continued)

ID
Reimherr1990 (SER 104 - FDA)
Doogan1994
Lydiard1997
Schneider2003
Moscovitch2004
Barber2011
Binnemann2008 (NCT00143091)
Brunoni2012
Fabre1995 (SER 103 FDA)
SER 315 (FDA)
Mao2015 (NCT01098318)
Davidson2002 (HDTSG) (NCT00005013)
Olie1997
PZ/109
PZ/111
Sheehan2009b (NCT00775203)
Rickels1982
Zhang2014
Cunningham1994 (VEN 600A-302 FDA)
VEN 600A-303 (FDA)
VEN 600A-313 (FDA)
VEN XR 367 (FDA)
Schweizer1994 (VEN 600A-301 FDA)
Guelfi1995 (VEN 600A-206 FDA)

(continued)

ID
Lecrubier1997
Cunningham1997 (VEN XR 208 - FDA)
Thase1997 (VEN XR 209 FDA)
Khan1998
Rudolph1998 (VEN 600A-203 (FDA)
Rudolph1999
Silverstone1999
Schatzberg2006a
Nemeroff2007
Sheehan2009a
Learned2012a Study1 (EUCTR2005-003401-87, SND103285, NCT00448058)
Higuchi 2014(NCT01441440)
Rickels2009 (GNSC-04-DP-02 FDA) (NCT00285376)
Khan2011 (CLDA-07-DP-02 FDA) (NCT00683592)
FDA 245 (EMD 68 843-010)
FDA 246 (SB 659746-003)
FDA 247 (SB 659746-014)
FDA 248 (SB 659746-002)
Croft2014 (NCT01473394)
Mathews2015 (NCT01473381)
Pomara2013
Boulenger2014 (13267A, NCT01140906)
Jacobsen2015 (316, NCT01163266)
Mahableshwarkar2015a (315, NCT01153009)

(continued)

ID
Mahableshwarkar2015b (317, NCT01179516)
NCT01355081(japicCTI-111492, U1111-1120-9277)
NCT01255787 (EUCTR2010-022257-41, Lu AA21004/CCT-002, U1111-1117-6595, JapicCTI-101344, CT
McIntyre2014 (NCT01422213)
Mahableshwarkar2015c (NCT01564862)
